# Determination of sample stability for a broad panel of coagulation parameters and factor assays on the Cobas t 711 analyzer starting from fresh-never-frozen plasma

**DOI:** 10.3389/fmolb.2025.1491239

**Published:** 2025-04-03

**Authors:** Michael Hoffmann, Norbert Gottschalk, Philipp Huber, Vanessa Scheling, Nicole von Allmen, J. Kolja Hegel

**Affiliations:** ^1^ Roche Diagnostics GmbH, Penzberg, Germany; ^2^ Roche Diagnostics International Ltd., Rotkreuz, Switzerland; ^3^ Labor Berlin – Charité Vivantes Services GmbH, Berlin, Germany

**Keywords:** blood coagulation, coagulation testing, fresh-never-frozen plasma, Cobas t 711 analyzer, sample stability, storage duration, temperature

## Abstract

**Background:**

Preanalytical procedures can affect the accuracy of coagulation assay results. Recommended plasma storage temperatures and durations need to be defined for individual coagulation assays. Here, we evaluated the effect of commonly applied plasma storage conditions for a broad panel of 23 basic coagulation parameters as well as specialized factor assays developed for the Cobas^®^ t 711 analyzer (Roche Diagnostics International Ltd., Rotkreuz, Switzerland).

**Methods:**

This single-center, prospective, observational study used anonymized, residual, platelet-poor plasma samples as well as pseudonymized plasma samples to obtain rare ranges of certain analytes. Fresh-never-frozen plasma samples processed within 4 h were tested in triplicate at time zero (t_0_), with measurements repeated at various predefined timepoints after storage at 18–25°C, 2–8°C, or under freezing and deep freezing. Mean deviation from t_0_, expressed as a percentage or as absolute change in signal at very low analyte levels, was assessed against predefined, assay-specific acceptance criteria for each analyte.

**Results:**

The sample stability results under the examined storage conditions for all 23 assays met or exceeded the requirements for routine laboratory coagulation testing and the respective acceptance criteria for each individual assay were fulfilled. Fresh-never-frozen samples were used to reflect real-life laboratory settings, enabling the early detection of out-of-specification results.

**Conclusion:**

Sample stability was determined for a broad panel of assays on the t 711 analyzer, for application in routine coagulation testing practice.

## Introduction

Coagulation assays are routinely used in hemostasis laboratories to screen, diagnose, and assess coagulopathies, and to monitor anticoagulant therapy (Winter et al., 2017; Adcock Funk, 2012). Given the importance of coagulation assays in clinical practice, it is essential that these analytical tests provide accurate and reliable results. However, preanalytical procedures have been found to affect the accuracy of coagulation test results, which may in turn affect patient diagnosis and monitoring of anticoagulant therapy (Favaloro et al., 2012; Magnette et al., 2016; Marlar and Rollins-Raval, 2019; Gosselin, 2021; Favaloro and Pasalic, 2024). As indicated in the Clinical and Laboratory Standards Institute guidelines, the results of coagulation tests can be influenced by many variables, such as sample stability under different storage conditions (time, temperature), indicating the need to determine the optimal storage temperature and acceptable time period for individual assays (Gosselin, 2021; Adcock et al., 2008; Feng et al., 2014; Gosselin et al., 2020; Toulon et al., 2017).

In this single-center, prospective, non-interventional, observational study, we aimed to validate stability claims for the registration of a broad panel of 23 coagulation assays on the Cobas® t 711 coagulation analyzer (Roche Diagnostics International Ltd., Rotkreuz, Switzerland) by evaluating the effect of different storage conditions of human fresh-never-frozen plasma samples on the assay measurements. To the best of our knowledge, this is the first time fresh-never-frozen plasma samples have been used to assess sample stability for coagulation parameters and factor assays on a medium- or high-throughput analyzer under conditions that reflect clinical reality, i.e., using samples that were collected and delivered to the laboratory fresh, without freezing, and then used immediately as starting point for the stability experiments.

## Methods

### Study design

A total of 23 basic routine parameters (D-Dimer Gen.2 [Tina-quant®; D-DI2], prothrombin time [PT rec]/prothrombin time-derived fibrinogen, thrombin time, antithrombin, fibrinogen, activated partial thromboplastin time [aPTT], aPTT screen, aPTT lupus, anti-Xa, Owrens prothrombin time [PT Owren]), more specialized tests (von Willebrand factor, lupus screen/confirm, free protein S, protein C), and factor assays (factors II, V, VII, VIII, IX, X, XI, XII, XIII) (all Roche Diagnostics International Ltd.) were assessed using the t 711 analyzer, a fully automated, high-throughput instrument designed for the *in vitro* qualitative and quantitative determination of coagulation analytes in human fresh-never-frozen plasma, with the aim of diagnosing coagulation abnormalities and monitoring anticoagulant therapy (Lippi et al., 2019; Roche Diagnostics GmbH, 2022; Roche Diagnostics GmbH, 2023; Roche Diagnostics GmbH, 2024a; Roche Diagnostics GmbH, 2024b; Roche Diagnostics GmbH, 2024c; Roche Diagnostics GmbH, 2024d; Roche Diagnostics GmbH, 2024e; Roche Diagnostics GmbH, 2024f; Roche Diagnostics GmbH, 2024g; Roche Diagnostics GmbH, 2024h; Roche Diagnostics GmbH, 2024i; Roche Diagnostics GmbH, 2024j; Roche Diagnostics GmbH, 2024k; Roche Diagnostics GmbH, 2024l; Roche Diagnostics GmbH, 2024m; Roche Diagnostics GmbH, 2024n; Roche Diagnostics GmbH, 2024o; Roche Diagnostics GmbH, 2024p; Roche Diagnostics GmbH, 2024q; Roche Diagnostics GmbH, 2024r; Roche Diagnostics GmbH, 2024s; Roche Diagnostics GmbH, 2024t; Roche Diagnostics GmbH, 2024u). The use of fresh-never-frozen plasma in this study reflects the storage conditions of routine laboratory settings, by collecting and delivering fresh samples to the laboratory. Samples were not frozen after collection; they were only frozen to verify the stability of analytes under certain conditions (−20°C, −80°C) after their levels had been determined at ambient temperature. The principles of the 23 assays used in this study are provided in Table 1.

**TABLE 1:** Principles of the 23 assays on the Cobas t 711 analyzer.

Assay	Principle
D-DI2 (Roche Diagnostics GmbH, 2022)	*In vitro* latex particle-enhanced immunoturbidimetric assay used for the quantitative immunologic determination of fibrin degradation products (D-dimer and X-oligomers) in human citrated plasma
PT rec/PT-der Fib (Roche Diagnostics GmbH, 2024r)	*In vitro* assay used to measure the time elapsed between the addition of thromboplastin and calcium to citrated human plasma, and the formation of a fibrin clot
Thrombin time (Roche Diagnostics GmbH, 2024s)	*In vitro* assay using a buffered thrombin reagent to measure the time from reagent addition to clot formation in human citrated plasma
Antithrombin (Roche Diagnostics GmbH, 2024t)	*In vitro* assay using excess amounts of heparin and thrombin to measure antithrombin activity in human citrated plasma
Fibrinogen (Roche Diagnostics GmbH, 2024m)	*In vitro* assay used for the quantitative determination of fibrinogen; the conversion time of soluble fibrinogen to insoluble fibrin polymers after the addition of excess thrombin is inversely proportional to fibrinogen concentration
aPTT (Roche Diagnostics GmbH, 2024a)	*In vitro* assays used to measure the time to clot formation upon addition of the aPTT reagent and calcium chloride in human citrated plasma
aPTT screen (Roche Diagnostics GmbH, 2024c)	
aPTT lupus (Roche Diagnostics GmbH, 2024b)	
vWF Act (Roche Diagnostics GmbH, 2024u)	*In vitro* particle-enhanced turbidimetric assay used to quantitatively measure platelet glycoprotein Ib binding vWF Act in human citrated plasma
Protein C (Roche Diagnostics GmbH, 2024q)	*In vitro* chromogenic assay used to quantitatively measure protein C activity in human citrated plasma
Free protein S (Roche Diagnostics GmbH, 2024n)	*In vitro* latex particle-enhanced turbidimetric assay used to quantitatively measure free protein S in human citrated plasma
Anti-Xa (Roche Diagnostics GmbH, 2023)	*In vitro* assay using heparin to quantitatively measure anti-Xa activity in human citrated plasma
PT Owren (Roche Diagnostics GmbH, 2024p)	*In vitro* assay used to measure the time to fibrin clot formation upon addition of the PT Owren reagent and calcium chloride in human citrated plasma
Lupus screen/confirm (Roche Diagnostics GmbH, 2024o)	*In vitro* assays used to screen for (lupus S) and confirm (lupus C) the presence of lupus anticoagulants by measuring the time to clot formation upon addition of diluted Russell’s viper venom reagent in human citrated plasma
Factor II (Roche Diagnostics GmbH, 2024d)	*In vitro* assays used to quantitively measure the activity of factor II, factor V, factor VII, and factor X, respectively, using a PT reagent and human citrated plasma immunodepleted of the respective factor
Factor V (Roche Diagnostics GmbH, 2024f)	
Factor VII (Roche Diagnostics GmbH, 2024g)	
Factor X (Roche Diagnostics GmbH, 2024i)	
Factor VIII (Roche Diagnostics GmbH, 2024h)	*In vitro* aPTT-type assays used to quantitively measure the activity of factor VIII, factor IX, factor XI, and factor XII, respectively, in human citrated plasma immunodepleted of the respective factor
Factor IX (Roche Diagnostics GmbH, 2024e)	
Factor XI (Roche Diagnostics GmbH, 2024j)	
Factor XII (Roche Diagnostics GmbH, 2024k)	
Factor XIII (Roche Diagnostics GmbH, 2024l)	*In vitro* latex particle-enhanced immunoturbidimetric assay used for the quantitative immunologic determination of factor XIII in human citrated plasma

aPTT, activated partial thromboplastin time; D-DI2, D-Dimer Gen.2 (Tina-quant); PT, prothrombin time; PT-der Fib, prothrombin time-derived fibrinogen; PT Owren, Owrens prothrombin time; PT rec, prothrombin time; vWF Act, von Willebrand factor activity.

Special effort was made to collect data reflecting the storage conditions of routine laboratory settings; in particular, fresh patient-derived plasma that had never been frozen was used as the starting point for all analyses. All fresh samples were collected from routine laboratories or specialized coagulation testing centers at Charité University Clinics in Berlin, Germany. No frozen samples (e.g., from vendors) were used as starting material at any point of the study.

Samples used in the analysis were required to contain analyte concentrations in the ranges defined by the study protocol and determined using routine methodology at the study site’s routine laboratory. The entire measuring range for each analyte was subdivided into up to six concentration groups, including the lowest, highest, and around the cut-off concentration values for each individual assay, and at least one sample per group was required to assess the effect of different storage conditions. Rare ranges were covered by samples containing either very low or very high analyte levels, both of which are typically harder to identify. Spiking and dilution were only used in a maximum of 10% of the samples in each experiment (in general, 1 out of 10), or at a maximum of 20% (2 out of 10) if clinicians had confirmed after several months of screening that such samples were very unlikely to be found in native form.

All experiments were conducted in a single laboratory using either anonymized, residual, platelet-poor plasma samples, which were left over from routine sample collection and previous testing performed at the same institution but in a different laboratory and by different personnel, independent from the study, or pseudonymized plasma samples from consenting donors recruited at specialized testing centers specifically for this study. Pseudonymized plasma samples from consenting donors were also used to obtain rare ranges of certain analytes, where necessary and applicable. Patient medication history was unknown for the anonymized leftover samples. However, for the pseudonymized donor samples, patients were excluded if they were taking anticoagulation medication, including but not limited to acetyl salicylic acid, direct oral anticoagulants, marcumar, and warfarin; or if they were pregnant and/or breast-feeding. In addition, visibly colored samples, such as lipemic, icteric, or hemolytic samples, were excluded from the analysis.

### Sample handling

Plasma samples were collected in 3.2% sodium citrate tubes, centrifuged twice at 2,500 *g* for 15 min, and sample aliquots up to 500 μL were tested within 4 h of collection (still unfrozen) to determine the time zero (t_0_) analyte levels. After the t_0_ analyte levels were specified, the sample aliquots (500 μL) were stored under different storage conditions (ambient temperature [18–25°C], refrigerated [2–8°C], freezing [–20°C], deep freezing [–80°C]), and tested in triplicate at predefined timepoints (Table 2). Based on applicable standard operating procedures, the last timepoint was one “time unit” (e.g., days) beyond the desired stability claim, except for −80°C testing. For a separate set of samples examined under freezing or deep freezing, aliquots were tested after one, two, and three freeze/thaw or deep freeze/thaw cycles; thawing was performed at room temperature (15–25°C).

**TABLE 2:** Assay results across timepoints and concentrations where the acceptance criteria were met, per storage condition.

Assay	Storage temperature	Timepoints	Determined stability	Concentration range	Acceptance criteria^a^	Mean deviation from t_0_ (%)^b^
D-DI2^c^ (Roche Diagnostics GmbH, 2022)	18–25°C	0, 6, 8, 12–18 h	8 h	0.2–9.0 μg FEU/mL	0.2–0.5 μg FEU/mL: ±0.05 μg FEU/mL^c^; >0.5 μg FEU/mL: ±10%	−2.7 to 0.9
	2–8°C	0, 6, 12, 24 h; 2, 4, 5 days	4 days			−6.3 to 0.9
	−20°C (short term)	0, 1, 2, 4, 5 weeks	28 days			−6.5 to 1.3
	−20°C (long term)	0, 1, 2, 4, 5 weeks, 3 months	11 weeks			−6.5 to 1.3
	Freeze/thaw	2 × 48 h	1×			−4.8 to –2.2
	Deep freeze/thaw	3 × 48 h	3×			−7.6 to 4.2
	−80°C (long term)	0, 3–7, 11–14, 17–21, 23–24 months	23 months			−6.6 to 6.3
PT rec (Roche Diagnostics GmbH, 2024r)	18–25°C	0, 10, 12, 13 h	12 h	0.8–5.0 INR	±10%	−4.5 to 7.9
	−20°C (long term)	0, 2, 4, 6, 8 weeks	7 weeks			−1.0 to 10.4
	Freeze/thaw	3 × 48 h	1×			0.5 to 6.0
	Deep freeze/thaw	3 × 48 h	1×			−5.7 to 0.5
	−80°C (long term)	0, 1, 3, 6, 12 months	11 months			−3.3 to 2.5
PT-der Fib (Roche Diagnostics GmbH, 2024r)	18–25°C	0, 6, 12–18, 24, 25 h	12 h	180–500 mg/dL	±10%	−7.1 to 0.9
	−20°C	0, 2, 4, 6, 8 weeks	7 weeks			−10.0 to 6.0
	Freeze/thaw	3 × 48 h	1×			−7.0 to −0.1
	Deep freeze/thaw	3 × 48 h	1×			−5.6 to 1.1
	−80°C (long term)	0, 1, 3, 6, 12–13 months	11 months			−5.0 to 1.0
Thrombin time (Roche Diagnostics GmbH, 2024s)	18–25°C	0, 1, 2, 4, 5 h	4 h	14–120 s	±10%; ±20% for heparin samples	−18.7^c^ to 1.5
Antithrombin (Roche Diagnostics GmbH, 2024t)	18–25°C	0, 12–18 h, 1, 2, 3 days	2 days	15–150%	≤40.0%: ±4.0% (absolute); >40.0%: ±10% (relative)	−8.1 to 4.3
	2–8°C	0, 3, 7, 8, 14, 15 days	14 days			−5.8 to 3.8
	−20°C (short term)	0, 1, 2, 3, 4, 5 weeks	28 days			−8.9 to 4.5
	−20°C (long term)	0, 1, 2, 3, 4, 5, 6, 8 weeks	7 weeks			−9.3 to 4.8
	Freeze/thaw	2 × 48 h	1×			−8.2 to 0.6
	Deep freeze/thaw	3 × 48 h	3×			−6.8 to 5.0
	−80°C	0, 4–8, 12–14, 15–19, 20–24 months	23 months			−5.9 to 4.1
Fibrinogen (Roche Diagnostics GmbH, 2024m)	18–25°C	0, 1, 2, 4, 5 h	4 h	60–900 mg/dL	±10%	−1.7 to 3.0
	−20°C	0, 2, 4, 6, 8 weeks	7 weeks			−8.0 to 5.0
	Freeze/thaw	2 × 48 h	1×			−8.1 to 2.7
	Deep freeze/thaw	3 × 48 h	1×			−13.9 to 0.6
	−80°C	0, 1, 3, 4, 6, 12 months	11 months			−9.5 to 10.8
aPTT (Roche Diagnostics GmbH, 2024a)	18–25°C	0, 1, 2, 4, 5 h	2 h	25–160 s	<100 s: ±10%; ≥100 s: ±20%	−0.5 to 16.6
	Deep freeze/thaw	3 × 48 h	1×			0.0 to 9.9
	−80°C	0, 3–4, 6–7, 12–13 months	11 months			−9.9 to 10.4
aPTT screen (Roche Diagnostics GmbH, 2024c)	18–25°C	0, 1, 2, 4, 5 h	2 h	25–160 s	<100 s: ±10%; ≥100 s: ±20%	−4.2 to 10.3
	Deep freeze/thaw	3 × 48 h	1×			−7.1 to 8.8
	−80°C	0, 3, 6–7, 12–13 months	11 weeks			−0.2 to 10.3
aPTT lupus (Roche Diagnostics GmbH, 2024b)	18–25°C	0, 1, 2, 4, 5 h	2 h	25–160 s	<100 s: ±10%; ≥100 s: ±20%	−4.2 to 9.8
	Deep freeze/thaw	3 × 48 h	1×			−4.2 to 7.2
	−80°C	0, 3–4, 6–7, 12–13 months	5 months			−8.5 to 9.6
vWF Act (Roche Diagnostics GmbH, 2024u)	18–25°C	0, 4, 5, 24–25 h	24 h	4–150%	±10%	−4.7 to 3.6
	−20°C	0, 4, 7, 8, 9, 12, 13 weeks	12 weeks			−14.4 to 12.1
	Freeze/thaw	3 × 48 h	1×			−7.4 to 9.8
	Deep freeze/thaw	3 × 48 h	3×			−2.4 to 13
	−80°C	0, 3–7, 8–12 months	3 months			−2.2 to 33.4
Protein C (Roche Diagnostics GmbH, 2024q)	18–25°C	0, 2, 3, 4, 5 h	4 h	4–150%	4.00–8.00%: ±0.800% (absolute); >8.00%: ±10.0% (relative)	−0.6 to 6.4
	−20°C	0, 7, 14, 28–29, 33–36 days	28 days			−2.9 to 4.8
	Freeze/thaw	2 × 48 h	1×			−4.7 to 5.8
	Deep freeze/thaw	3 × 48 h	3×			−3.7 to 3.8
	−80°C	0, 6, 9, 10–18, 23–25 months	10 months			−1.5 to 6
Free protein S (Roche Diagnostics GmbH, 2024n)	18–25°C	0, 8, 10, 11, 24, 25 h	24 h	10–150%	<20.0%: ≤±2.00% (absolute); ≥20.0%: ≤±10.0% (relative)	−5.9 to 2.7
	−20°C	0, 4, 7, 8, 9, 12, 13 weeks	12 weeks			−9.5 to 1
	Freeze/thaw	2 × 48 h	1×			−7.6 to 1.8
	Deep freeze/thaw	3 × 48 h	3×			−6.7 to 0.733
	−80°C	0, 6–7, 10–12, 18–20, 23–25 months	24 months			−4.1 to 5.1
Anti-Xa (Roche Diagnostics GmbH, 2023)	18–25°C	0, 1, 2, 3, 4, 5 h	2 h	0.10–2.00 IU/mL	≤0.45 IU/mL: ≤0.045 IU/mL; >0.45 IU/mL: ≤10%	−8.8 to 3.7
	−20°C	0, 14, 15, 28, 29 days	14 days			−10.7 to 7.2
	Freeze/thaw	2 × 48 h	1×			−9.8 to 4.3
	Deep freeze/thaw	3 × 48 h	3×			−2.6 to 4.6
	−80°C (long term)	0, 1–3, 4–5, 6–7 months	11 weeks			−13.1 to 8.8
PT Owren (Roche Diagnostics GmbH, 2024p)	18–25°C	0, 6, 12–18, 24, 25 h	24 h	0.8–>6.0 INR	≤4.5 INR: 100% ± 10%; >4.5 INR: 100% ± 15%	−8.4 to 2.1
	−20°C	0, 2–5, 6–7, 8–9 weeks	6 weeks			−1.5 to 10.4
	Freeze/thaw	2 × 48 h	1×			−0.1 to 6.1
	Deep freeze/thaw	3 × 48 h	1×			−1.2 to 3.5
	−80°C	0, 1, 3–6, 12–14 months	3 months			−0.7 to 7.7
Lupus screen/confirm (Roche Diagnostics GmbH, 2024o)	18–25°C	0, 2, 3, 4, 5 h	4 h	≤1.20–>1.80 INR	±10.0% up to INR 1.5; ±20.0% for INR ≥1.5 based on seconds	−7.8 to 17.7
	Deep freeze/thaw	3 × 48 h	1×			−2.5 to 9.6
	−80°C	0, 7, 14, 15, 28, 29 days, 12–13 weeks	12 weeks			−6.6 to 8.5
Factor II (Roche Diagnostics GmbH, 2024d)	18–25°C	0, 2, 3, 4, 5 h	4 h	2–150%	±15%	−5.2 to 2.2
	−20°C	0, 7, 8, 14, 15, 28, 29 days	28 days			−15.9 to 3.9
	Freeze/thaw	2 × 48 h	1×			−3.7 to 0.9
	Deep freeze/thaw	3 × 48 h	3×			−0.5 to 2.6
	−80°C	0, 3, 6, 12, 18, 24 months	12 months			−16.6 to 5.3
Factor V (Roche Diagnostics GmbH, 2024f)	18–25°C	0, 2, 3, 4, 5 h	4 h	1.5–150%	±15%	−8.3 to 3.9
	Deep freeze/thaw	3 × 48 h	1×			−22.8 to 5.4
	−80°C (short term)	0, 7, 8, 14, 15, 28, 29 days	28 days			−5.5 to 11.6
	−80°C (long term)	0, 3, 6, 12, 18, 24 months	24 months			−15.8 to 13.5
Factor VII (Roche Diagnostics GmbH, 2024g)	18–25°C	0, 2, 3, 4, 5 h	4 h	1.5–200%	±15%	−0.7 to 13.2
	−20°C	0, 7, 8, 14, 15, 28, 29 days	28 days			−5.8 to 3.2
	Freeze/thaw	2 × 48 h	1×			−3.8 to 856.9
	Deep freeze/thaw	3 × 48 h	3×			−1.4 to 12.2
	−80°C	0, 3, 6, 12, 18, 24 months	24 months			−8.9 to 11.6
Factor VIII (Roche Diagnostics GmbH, 2024h)	18–25°C	0, 1, 1.3, 2, 2.3, 3 h	2 h	0.2–150%	±15%	−22.8 to 4.7
	−20°C	0, 7, 8, 14–17, 28–36 days	14 days			−26 to −4.4
	Freeze/thaw	3 × 48 h	1×			−26.3 to 11.7
Factor IX (Roche Diagnostics GmbH, 2024e)	18–25°C	0, 2, 3, 4, 5 h	4 h	0.25–150%	≤1.25%: ±0.250% (absolute); >1.25–≤30.0%: ±20.0% (relative); >30.0%: ±15.0%	−10.3 to 3.6
	−20°C	0, 7, 8, 14, 15, 28, 29 days	14 days			−17.3 to 4
	Freeze/thaw	3 × 48 h	1×			−12.9 to 1.9
	Deep freeze/thaw	3 × 48 h	3×			−9.5 to 2.5
	−80°C (short term)	0, 7, 8, 14, 15, 28, 29 days	28 days			−11 to 9.2
	−80°C (long term)	0, 1, 3, 6, 12, 18, 24 months	24 months			−14.3 to 45.4
Factor X (Roche Diagnostics GmbH, 2024i)	18–25°C	0, 2, 3, 4, 5 h	4 h	1.0–200%	±10%	−1.2 to 4.2
	−20°C	0, 7, 14, 15, 28, 29 days	28 days			−8.4 to 6.5
	Freeze/thaw	2 × 48 h	1×			−4.1 to 7
	Deep freeze/thaw	3 × 48 h	1×			−5.5 to 10.4
	−80°C	0, 6, 12, 18, 24 months	24 months			−6 to 8.8
Factor XI (Roche Diagnostics GmbH, 2024j)	18–25°C	0, 2, 4, 5 h	4 h	1.0–150%	±15%	−14.5 to 0.6
	−20°C	0, 7, 8, 14, 15, 28, 29 days	28 days			−11 to 6.2
	Freeze/thaw	3 × 48 h	1×			−12.8 to 3.2
	Deep freeze/thaw	3 × 48 h	3×			−10.9 to 1.8
	−80°C (short term)	0, 7, 8, 14, 15, 28, 29 days	–			−8.7 to 3.9
	−80°C (long term)	0, 1, 3, 6, 12, 18, 24 months	24 months			−15.3 to 9.2
Factor XII (Roche Diagnostics GmbH, 2024k)	18–25°C	0, 2, 3, 4, 5 h	4 h	1.5–150%	±15%	−9.5 to 9.4
	−20°C	0, 7, 8, 14, 15, 28, 29 days	28 days			−14.3 to 11.3
	Freeze/thaw	3 × 48 h	1×			−14.7 to 13
	Deep freeze/thaw	3 × 48 h	3×			−10.1 to 9.9
	−80°C (short term)	0, 7, 8, 14, 15, 28, 29 days	28 days			−9.5 to 6.4
	−80°C (long term)	0, 1, 3, 6, 13–18 months	6 months			−12.1 to 21.2
Factor XIII (Roche Diagnostics GmbH, 2024l)	18–25°C	0, 2, 3, 4, 5 h	4 h	<9–109%	1.57–20.0% FXIII antigen potency: ≤±3.00% (absolute); >20.0–109% FXIII antigen potency: ≤±15.0% (relative)	−1.6 to 6.3
	−20°C	0, 7, 8, 14, 15, 28, 29 days	28 days			−3.9 to 9
	Freeze/thaw	3 × 48 h	1×			−2 to 3.4
	Deep freeze/thaw	3 × 48 h	3×			−2.6 to 3.6
	−80°C (short term)	0, 7, 8, 14, 15, 28, 29 days	28 days			–
	−80°C (long term)	0, 1, 3, 6, 11–14, 18, 24 months	24 months			−16.3 to 9.8

^a^ Indicated ± values are the maximum allowable mean deviation from t_0_ (relative difference [%] unless otherwise stated). t_0_ was always determined using a fresh-never-frozen plasma sample.

^b^ Observed range in mean deviation from t_0_ (minimum to maximum) across all concentrations tested, at timepoints where the acceptance criteria were still met and stability could therefore be deduced.

^c^ Heparinized sample for which a 20% drift from t_0_ is allowed as an acceptance criterion.

aPTT, activated partial thromboplastin time; D-DI2, D-Dimer Gen.2 (Tina-quant); FEU, fibrinogen equivalent unit; INR, international normalized ratio; IU, international units; PT-der Fib, prothrombin time-derived fibrinogen; PT Owren, Owrens prothrombin time; PT rec, prothrombin time; t_0_, time zero; vWF Act, von Willebrand factor activity.

For each experiment, at least 10 samples were tested and at least one sample was included in each of the predefined concentration ranges per experiment. For the freeze/thaw and deep freeze/thaw cycle experiments, a total of five samples (one sample for each concentration range) were tested. The temperatures and time of storage as well as the concentration ranges (covering the relevant measuring range) and acceptance criteria (% deviation of mean measurements from t_0_) for each assay by storage condition are summarized in Table 2.

### Statistical analysis and data management

The raw triplicate results (raw values, mean, median, standard deviation [SD], % coefficient of variation) obtained at each predefined storage timepoint were calculated for each sample and assay tested (line data not shown). In this study, for each storage condition tested, data are presented as the mean deviation from t_0_, which was calculated using the mean of triplicate measurements and assessed against predefined, assay-specific acceptance criteria for each analyte (Roche Diagnostics GmbH, 2022; Roche Diagnostics GmbH, 2023; Roche Diagnostics GmbH, 2024a; Roche Diagnostics GmbH, 2024b; Roche Diagnostics GmbH, 2024c; Roche Diagnostics GmbH, 2024d; Roche Diagnostics GmbH, 2024e; Roche Diagnostics GmbH, 2024f; Roche Diagnostics GmbH, 2024g; Roche Diagnostics GmbH, 2024h; Roche Diagnostics GmbH, 2024i; Roche Diagnostics GmbH, 2024j; Roche Diagnostics GmbH, 2024k; Roche Diagnostics GmbH, 2024l; Roche Diagnostics GmbH, 2024m; Roche Diagnostics GmbH, 2024n; Roche Diagnostics GmbH, 2024o; Roche Diagnostics GmbH, 2024p; Roche Diagnostics GmbH, 2024q; Roche Diagnostics GmbH, 2024r; Roche Diagnostics GmbH, 2024s; Roche Diagnostics GmbH, 2024t; Roche Diagnostics GmbH, 2024u). The maximum and minimum mean deviations obtained under storage conditions that still allowed to meet the respective acceptance criteria for each assay are provided in Table 2.

Acceptance criteria were defined as the level of changes allowed in the concentration or test signal of each analyte in samples measured after storage under the indicated conditions. These criteria were determined based on the experience of users who had measured the same analytes on other commercially available analyzers and who had informed about the concentration changes that can be considered acceptable in a routine laboratory setting.

Assay results were directly captured by WinCAEv, CFR 21 Part 11 compliant electronic data capture software and reviewed by the investigator for completeness and potential discrepancies.

## Results and discussion

This study validated stability claims for the registration of a broad panel of 23 coagulation assays on the t 711 coagulation analyzer, and showed that the examined storage times and conditions were acceptable and either satisfied or exceeded the requirements for routine laboratory testing, as judged by the investigators and representatives of routine testing laboratories (Table 2). The respective acceptance criteria were fulfilled at each timepoint measured, up to the claimed stability timepoints and at least one beyond, while long-term experiments were not continued beyond the indicated maximum timepoints. If, for a certain timepoint reached in the course of an experiment, the acceptance criteria were no longer met, it was concluded that the stability could only be claimed up to an earlier timepoint where results still met the criteria. As examples, for thrombin time, aPTT, lupus screen/confirm, and factor VIII, some of the initially assumed stability claims were not met or had to be reduced, demonstrating that the stability of fresh samples cannot be inferred from literature data but can only be determined if data are generated using the respective assays for a specific system. These findings also suggested that tests with a similar principle but from a different provider cannot be used to determine sample stability due to differences in antibodies between assays, as well as to antigen-antibody interactions that may have different sensitivity and susceptibility to changes occurring during storage.

The analytical performance for the t 711 assays was previously assessed in separate studies (Bertsch et al., 2023; Lowe et al., 2022; Kitchen et al., 2020; Kitchen et al., 2018a; Kitchen et al., 2018b). For example, the factor VIII assay demonstrated robust analytical performance on the t 711 analyzer; SD for repeatability was 0.016–0.046 for samples with ≤1.0 international units (IU)/dL of factor VIII activity (Lowe et al., 2022). In a simulated real-life laboratory setting, the t 711 analyzer demonstrated high performance and throughput across a variety of assays tested, including aPTT, aPTT lupus, aPTT screen, antithrombin, D-DI2, fibrinogen, prothrombin time-derived fibrinogen, PT Owren, PT rec, and thrombin time. Coefficients of variation ranged between 0.0–1.5% for intermediate precision, 0.2–3.0% for repeatability, and 0.4–3.6% for total precision (Kitchen et al., 2020). For all previously examined assays, analytical performance was within the specifications used in the present study.

In the current analysis, examining plasma samples that have been stored at ambient temperature (18–25°C), refrigerated (2–8°C), and/or under freezing (−20°C) and deep freezing (−80°C) at different timepoints indicated that correct handling procedures are required to ensure the accuracy of coagulation analyses with the t 711 analyzer. Since deep freezing (storage below −80°C) is not routinely applied in coagulation tests, the stability of samples under this condition was tested for internal purposes only or in the rare cases where stability at −20°C could not be demonstrated (aPTT, lupus screen/confirm). Optimal storage conditions vary depending on the analyte and the reason for storage, e.g., if samples need to be stored for repeated measurements or confirmatory testing, for weekly measurements, or for long-term scientific studies. For this study, which was conducted in a research laboratory setting, as a conservative approach and in order to achieve long-term stability, it is recommended that samples are stored at −80°C, which reflect the common storage conditions in research settings and all analytes are expected to be stable for 3–12 months (depending on the analyte). However, such low-temperature freezers are not always available in clinical routine testing laboratories; therefore, it is recommended to use the next best available storage conditions, e.g., storage at −20°C for 2–12 weeks depending on the analyte, especially if the collected samples are to be tested within a few days or weeks after collection and refrigeration at 4°C does not provide enough stability.

The key strength of this study is that only fresh-never-frozen samples across a wide range of analyte levels were used at the beginning of testing, using the initial t_0_ value as the reference point, which meets current requirements for market approval of coagulation assays by regulatory authorities, including the U.S. Food and Drug Administration. In addition, spiking and dilution were only applied when samples with relevant analyte levels could not be obtained otherwise. In such cases, the investigators aimed to use contrived samples only up to a limit of 10% of the total sample number in an experiment. Furthermore, the use of fresh-never-frozen samples instead of isochronic sample collection allowed the early detection of out-of-specification results. The availability of such data for the t 711 analyzer represents an advantage for its users, as to our knowledge, data acquired under clearly defined conditions in fresh-never-frozen plasma are currently not available for similar coagulation assay systems.

## Conclusion

In conclusion, we determined the stability for samples measured with the entire panel of relevant routine and more specialized factor assays on the t 711 analyzer, for coagulation analyte testing in routine laboratory settings. The use of fresh-never-frozen samples in this study provides increased confidence in the analytical results and recommended storage conditions for these assays, and to the best of our knowledge, this is the first use of such an approach.

## Data Availability

The original contributions presented in the study are included in the article. Requests concerning the data supporting the findings of this study can be directed to rotkreuz.datasharingrequests@roche.com.

## Abbreviations

aPTT, activated partial thromboplastin time; D-DI2, D-Dimer Gen.2 (Tina-quant); FEU, fibrinogen equivalent unit; INR, international normalized ratio; IU, international units; PT-der Fib, prothrombin time-derived fibrinogen; PT Owren, Owrens prothrombin time; PT rec, prothrombin time; SD, standard deviation; t_0_, time zero; vWF Act, von Willebrand factor activity.
